# Supplementary material for: High throughput estimation of functional cell activities reveals disease mechanisms and predicts relevant clinical outcomes
Source: Oncotarget. 2016 Dec 22;8(3):5160–78. doi: 10.18632/oncotarget.14107 (PMC5354899; doi:10.18632/oncotarget.14107)
Supplement: Supplementary file 1 [file oncotarget-08-5160-s001.pdf]

High throughput estimation of functional cell activities reveals disease mechanisms and predicts relevant clinical outcomes

SUPPLEMENTARY FIGURES AND TABLES

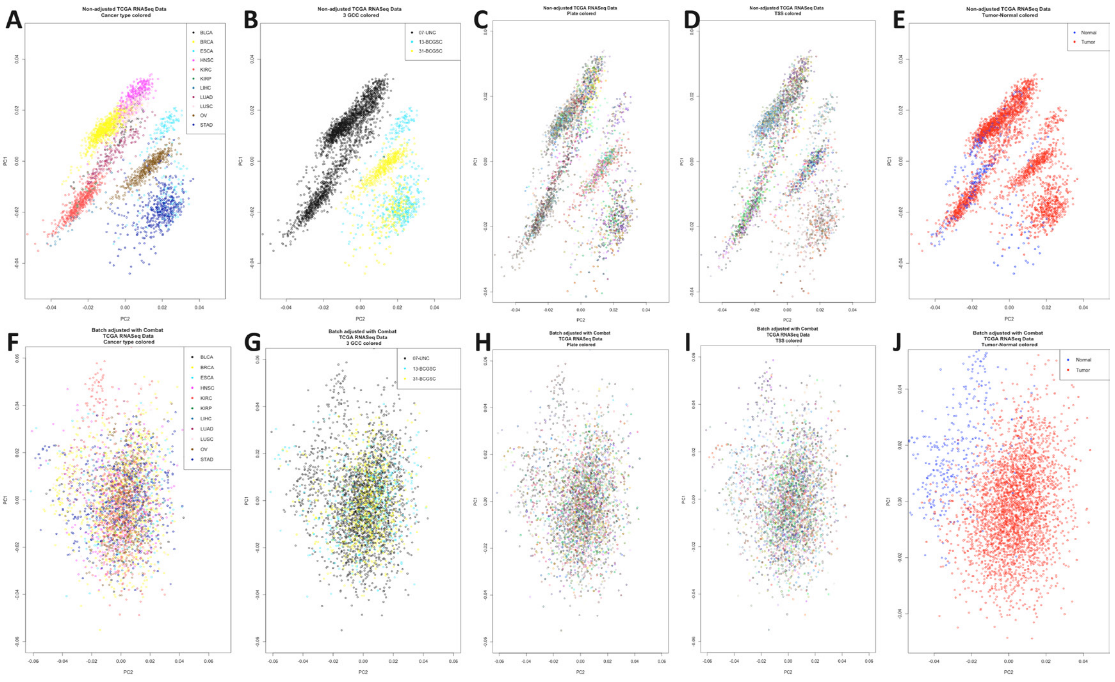

Figure S1. PCA plots of the samples to discover batch effects. Original samples colored by: **A.** cancer type, **B.** GCC, showing a clear batch effect in this case, **C.** plate, **D.** TSS and **E.** normal-tumor. Samples after batch effect correction colored by: **F.** cancer type, **G.** GCC, with no batch effect now, **H.** plate, **I.** TSS and **J.** normal-tumor, showing now a difference that can be attributed to the biology instead to the technology.

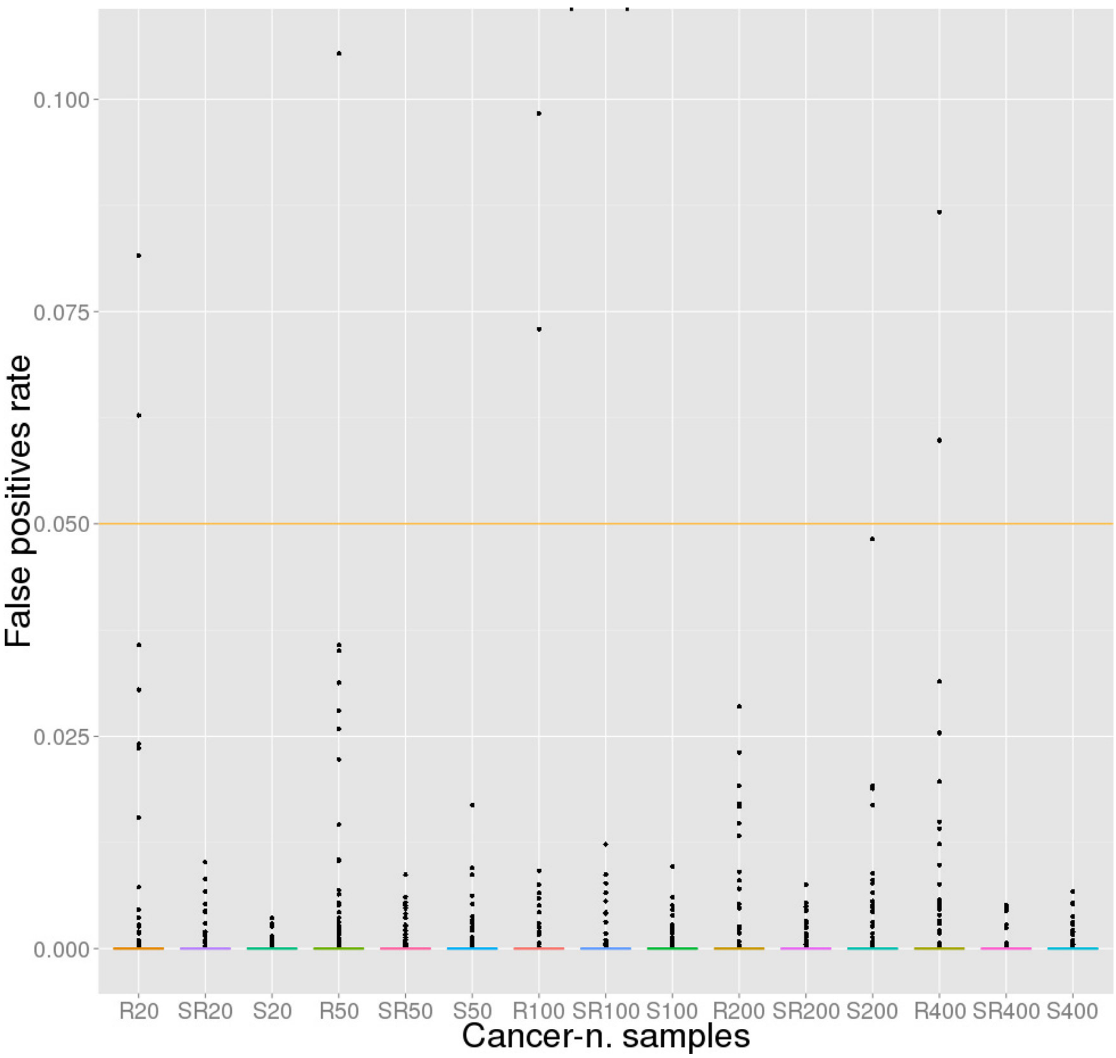

Figure S2. False positive ratio of the CCAA method proposed, obtained as the proportion of signaling circuits that present significant differential activity when identical datasets are compared. The identical datasets were generated by three methods: by randomly sampling KIRC patients in two groups of growing size (r label in the X axis), by simulating individuals with gene expression values taken from the average values observed in KIRC patients (label sr in the X axis) and by simulating individuals with gene expression values taken from a normal distribution N(0.5, 0.05) (label s in the X axis) (See Methods). The red line in the plot represents the conventional α value of 0.05. The X axis represents growing dataset sizes obtained under the three conditions above mentioned.

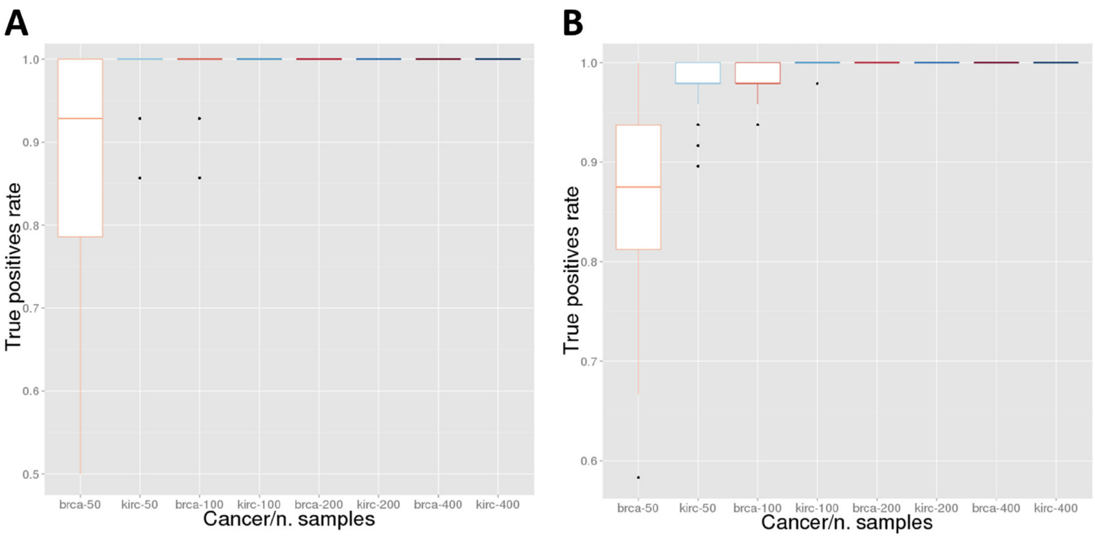

Figure S3. True positive ratio of CCAA method proposed obtained as the proportion of cancer pathways with one or more signaling circuits with a significant differential activity found by comparing cancer cases to their corresponding normal tissue samples, for which real differences are expected. Two sets of cancer pathways of reference are used: **A.** 14 KEGG cancer (Cancer pathways category, see Table S9) and **B.** 49 curated cancer pathways (see Table S10). The calculations were made for two cancers: BRCA and KIRC. Labels in the X axis correspond to growing sizes of datasets compared for both cancers.

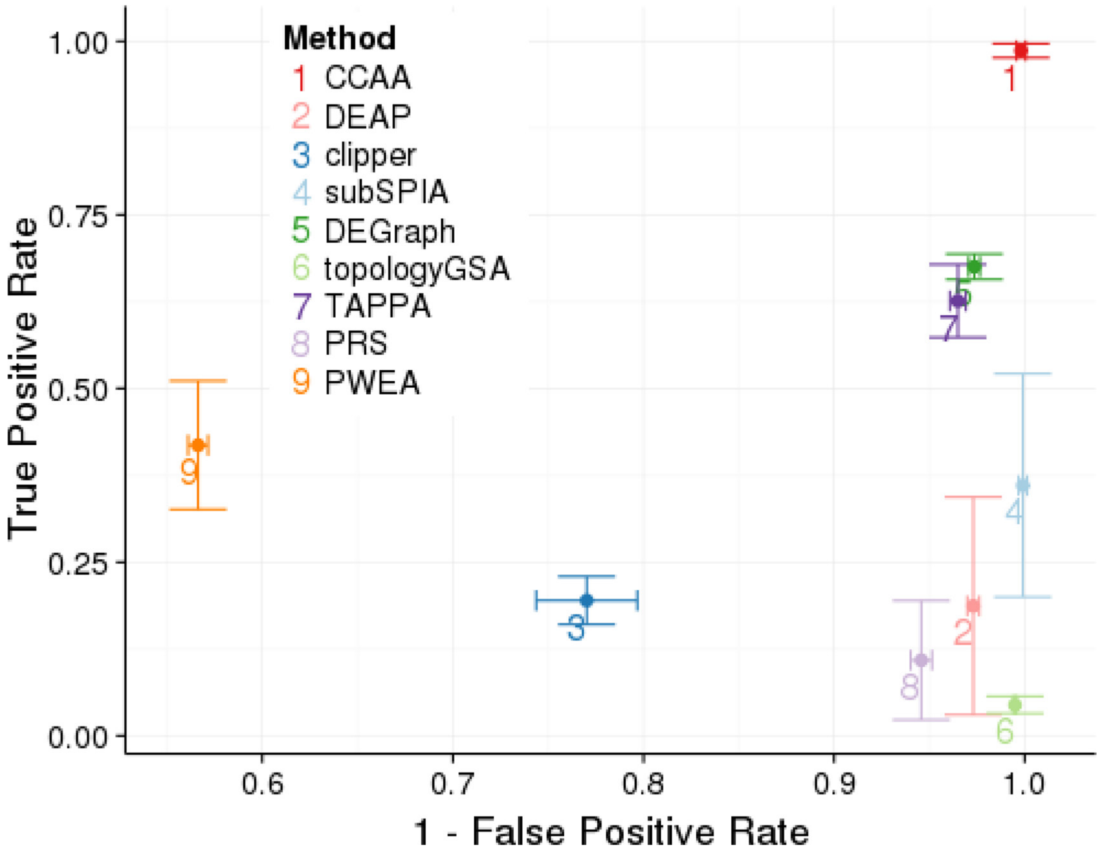

Figure S4. Comparison of performances of the different methods for defining pathways and calculating its activity. The true positive rate has been estimated averaging the proportion of significant curated cancer pathways (Table S10) across the 12 cancers analyzed and is represented in the Y axis. Vertical bars in each point represent 1 SD of the true positive rate for the corresponding method. The false positive rate was estimated from 100 comparisons of groups (N=25) of identical individuals, randomly sampled from each cancer. The results obtained in the 12 cancers are used to obtain a mean value and an error. The X axis represents 1- the false positive rate. Horizontal bars represent in each point represent 1 SD of the false positive rate for the corresponding method.

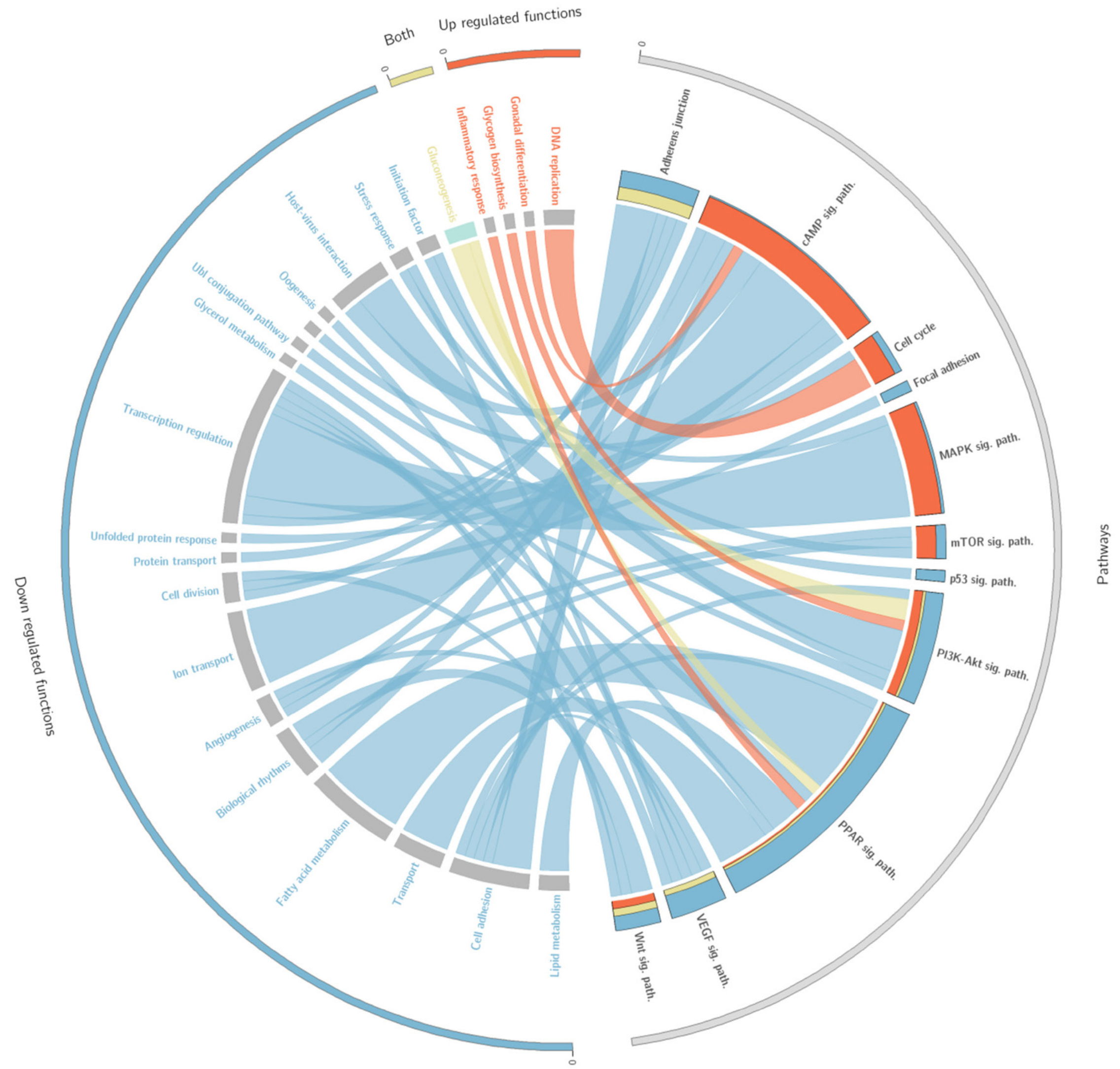

Figure S5. Circos plot that summarises the relationships between effectors within pathways and the functions triggered by them. Only curated cancer pathways (Table S10) related to functions significantly related to survival are represented here. On the right side appear the effector circuits grouped by pathway. There is a histogram per pathway that represents the proportion of effector pathways upregulated (red), downregulated (blue) and both regulation statuses (yellow). On the left side of the circo appear the functions triggered by the effector circuits divided into those which are significant when up-regulated (red), when down-regulated (blue) or when both situations can occur (yellow). For each function there is a thick line that indicates the prognostic of its deregulation, which can be good (green) or bad (grey).

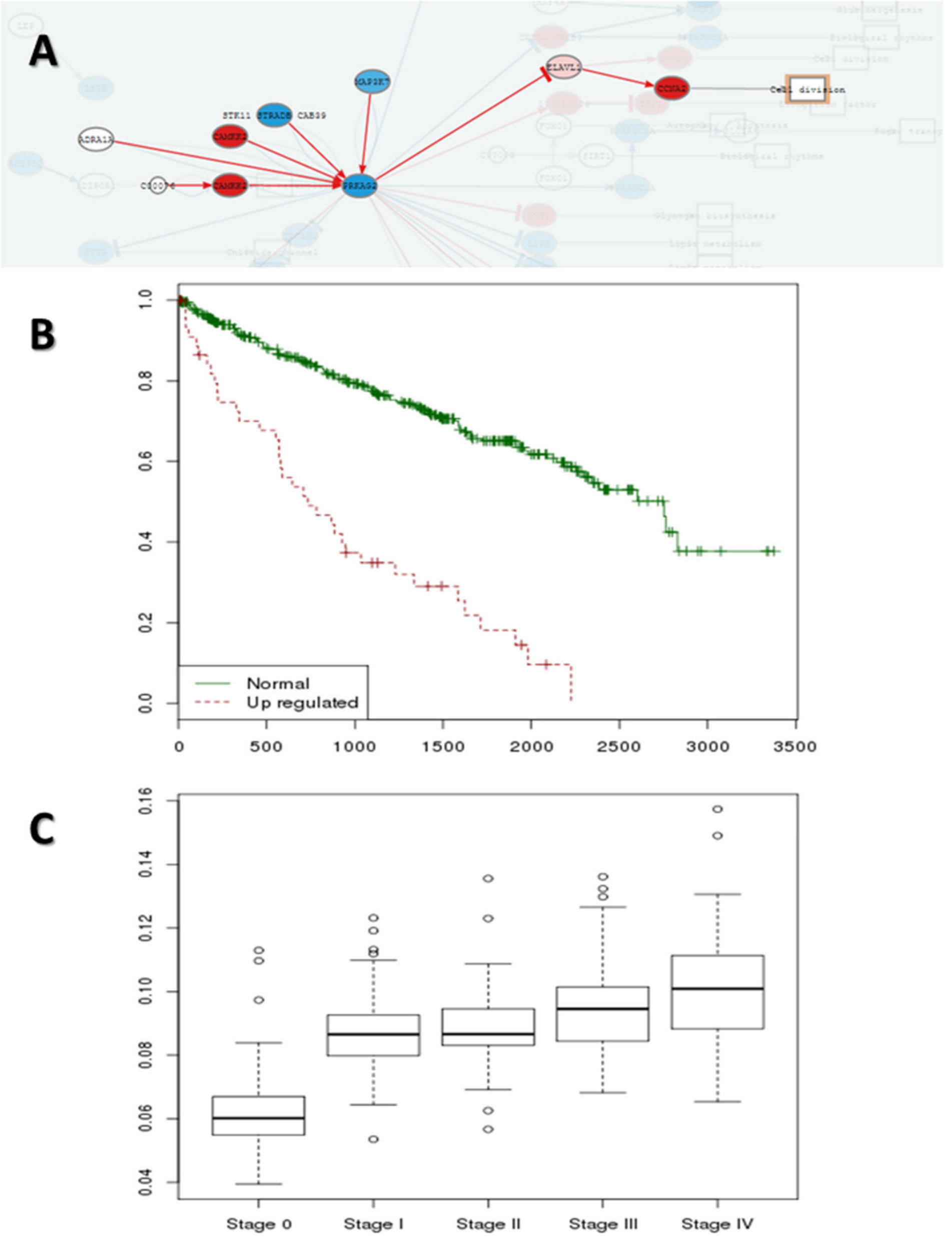

Figure S6. Example of effector circuit significantly associated to bad prognostic in KIRC. A. A detail of the AMPK signaling pathway with the effector pathway ending in the *CCNA2* protein that trigger *Cell division* is highlighted. The visualization was generated with the hipathia program (http://hipathia.babelomics.org). In the plot, arrows connecting the nodes appear in red, indicating that the effector circuit is activated. Also, expression changes of individual genes are represented: genes in blue are under-expressed in KIRC with respect to the normal kidney tissue while genes in red are over-expressed. **B.** Kaplan-Meyer curve demonstrating a significant association of high levels of activity in the effector circuit and a bad prognostic in the patients. **C.** Significant increase in the activity of the *CCNA2* effector circuit as cancer stage progresses.

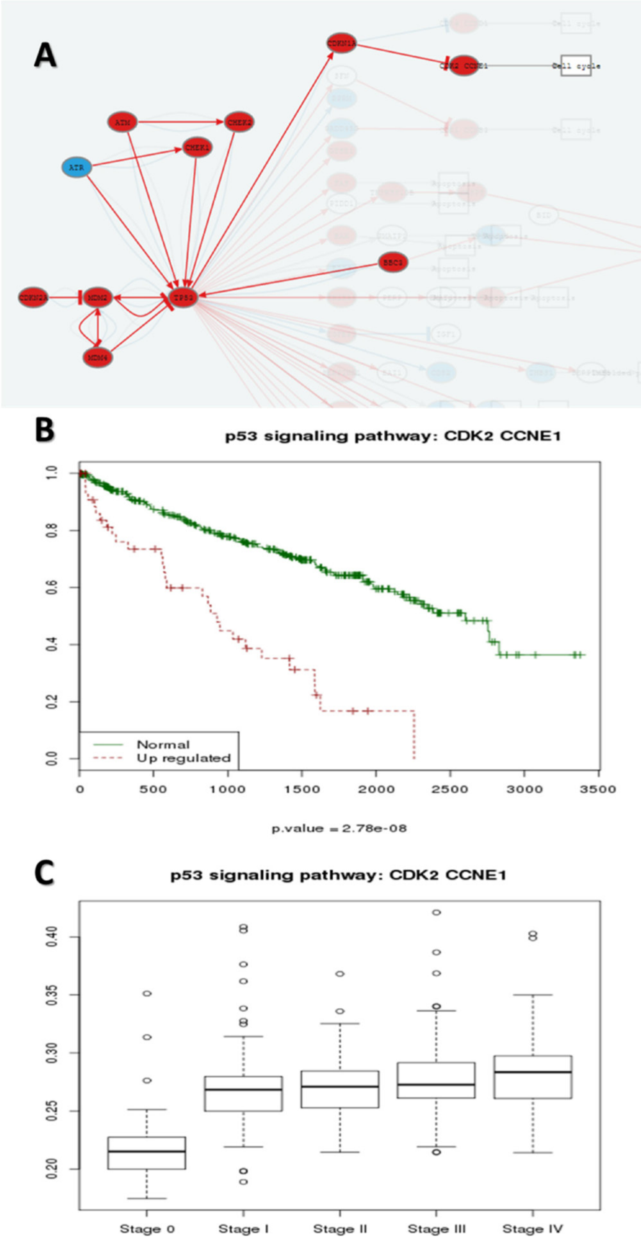

Figure S7. Example of effector circuit significantly associated to bad prognostic in KIRC. A. A detail of the p53 Signaling pathway with the effector pathway ending in the node containing proteins *CDK2* and *CCNE1* that trigger *Cell cycle* is highlighted. The visualization was generated with the hipathia program (http://hipathia.babelomics.org). In the plot, arrows connecting the nodes appear in red, indicating that the effector circuit is activated. Also, expression changes of individual genes are represented: genes in blue are under-expressed in KIRC with respect to the normal kidney tissue while genes in red are over-expressed. **B.** Kaplan-Meyer curve demonstrating a significant association of high levels of activity in the effector circuit and a bad prognostic in the patients. **C.** Significant increase in the activity of the *CDK2*, *CCNE1* effector circuit as cancer stage progresses.

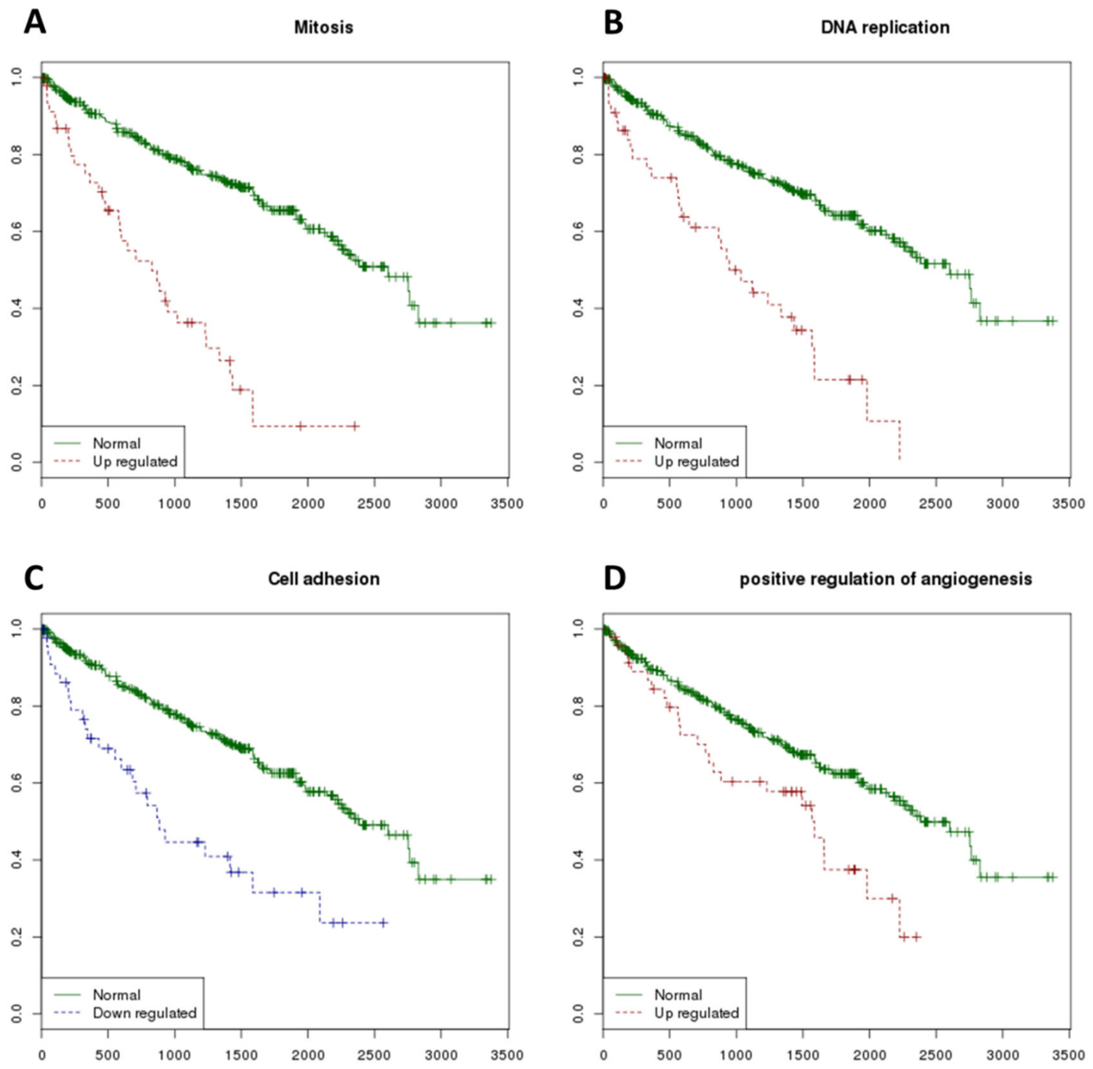

Figure S8. Survival Kaplan-Meier (K-M) curves obtained for Uniprot and GO functions. A. Mitosis and **B.** DNA replication, whose upregulation shows a significant association to increased mortality (FDR-adjusted p-values of 1.7×10^{-12} and 5.9×10^{-8}, respectively); **C.** Cell Adhesion, whose downregulation is significantly associated to bad prognostic (FDR-adjusted p-value=4.4×10^{-5}); **D.** Positive regulation of angiogenesis (FDR-adjusted p-value=2.9×10^{-2}).

Supplementary Table S1: Canonical circuits differentially activated between cancer and the normal tissue

See Supplementary File 1

Supplementary Table S2: Effector circuits differentially activated between cancer and the normal tissue

See Supplementary File 2

Supplementary Table S3: Unitprot functions differentially activated between cancer and the normal tissue

See Supplementary File 3

Supplementary Table S4: Gene Ontology functions differentially activated between cancer and the normal tissue

See Supplementary File 4

Supplementary Table S5: Effector circuits associated to patient survival

See Supplementary File 5

Supplementary Table S6: Uniprot functions associated to patient survival

See Supplementary File 6

Supplementary Table S7: Gene Ontology functions associated to patient survival

See Supplementary File 7
